# Insights into the potential pathogenesis and therapeutic implications of ferroptosis in brain microvascular endothelial cells during stroke

**DOI:** 10.3389/fimmu.2026.1787418

**Published:** 2026-02-26

**Authors:** Wenxiu Qin, Yiran Zhao, Jianqiang Du, Qiaoli Zhang, Gang Wei, Shaokang Wang, Ziru Yu, Junfeng Xu, Jian Yang, Ying Gao

**Affiliations:** 1 Acupuncture Department, First Teaching Hospital of Tianjin University of Traditional Chinese Medicine, Tianjin, China; 2 National Clinical Research Center for Chinese Medicine, Tianjin, China; 3 Graduate School, Tianjin University of Traditional Chinese Medicine, Tianjin, China; 4 Acupuncture Department, The Second Affiliated Hospital of Gansu University of Traditional Chinese Medicine, Lanzhou, China; 5 Department of Psychosomatic Medicine, First Teaching Hospital of Tianjin University of Traditional Chinese Medicine, Tianjin, China

**Keywords:** blood-brain barrier, brain microvascular endothelial cells, ferroptosis, mechanisms, stroke

## Abstract

Brain microvascular endothelial cells (BMECs) constitute the core component of the Blood-Brain Barrier (BBB), whose structural and functional integrity is crucial for maintaining central nervous system homeostasis. In recent years, ferroptosis—a novel iron-dependent lipid peroxidation-driven cell death pathway—has been demonstrated to play a pivotal role in secondary brain injury following stroke. However, current research predominantly focuses on ferroptosis in neurons and glial cells, with insufficient attention given to the mechanisms underlying BMEC ferroptosis in stroke pathogenesis. This review systematically examines the pivotal role of BMEC ferroptosis in the development of both ischemic and hemorrhagic strokes, elucidating its multiple pathways for exacerbating brain injury: compromising BBB integrity, triggering vasogenic cerebral edema, intensifying neuroinflammation, and promoting hemorrhagic transformation. The article highlights the molecular mechanisms of signaling pathways—including Meg3/p53/GPX4, TEAD1/MMP3, SESN2/System Xc−/GPX4, and SP1/TNFSF9/SLC3A2—in regulating BMEC ferroptosis. It summarizes multidimensional therapeutic strategies encompassing iron chelators, genetic/molecular interventions (e.g., FGF2, p23, METTL3, lncRNA H19), novel nanodelivery systems (e.g., RosA-LIP), and selenium compounds (SeMC). This study aims to provide new insights into vascular unit injury after stroke and establish theoretical foundations and translational directions for developing neuroprotective therapies targeting ferroptosis in BMECs.

## 1 Introduction

Stroke, which primarily includes ischemic stroke (IS) and hemorrhagic stroke (HS), has increased in absolute incidence and prevalence by 70% and 85%, respectively, according to the Global Burden of Disease Study 2019 (1). There were 3.94 million new stroke cases and 2.19 million stroke deaths in China in 2019 (2), suggesting that the Chinese population is at great risk of stroke. As a result, stroke remains the leading cause of death and disability, and the global burden of stroke remains high (3). IS characterized by occlusion of cerebral arteries or arteries supplying brain tissue accounts for about 82.6% of all types of stroke (4). Currently, the primary treatment for IS is prompt restoration of blood supply to the ischemic area. Thrombolysis and thrombolysis are the only two guideline-recommended treatments for IS, and are indicated within 24 hours of an attack (5). However, in patients with acute ischemic stroke undergoing intravenous thrombolysis or endovascular thrombectomy, revascularization may cause severe cerebral ischemia-reperfusion injury (CIRI) due to inflammatory response, reactive oxygen species (ROS) accumulation and excitotoxicity (6–8), and may also increase the risk of hemorrhagic transformation (9, 10), leading to irreversible neurological damage (11), worsening brain function and resulting in a poorer prognosis. HS includes cerebral hemorrhage (ICH) and subarachnoid hemorrhage (SAH), and HS is an important public health problem with high morbidity and mortality worldwide (12, 13). Despite the current major advances in the prevention and clinical management of ICH and SAH, global morbidity and associated mortality have increased over the past few decades (14, 15). Due to the high mortality and disability rates of HS, most survivors are left with sequelae such as motor, cognitive, speech or swallowing disorders to varying degrees, causing heavy care and financial burdens on society and families (16). Therefore, there is an urgent need to find new strategies for the treatment of stroke that address this issue in a clinical context.

It has been shown that ferroptosis in brain microvascular endothelial Cells (BMECs) mediates the pathophysiologic process of stroke, and inhibition of ferroptosis in BMECs may improve the prognosis of stroke (17, 18). Therefore, this review systematically summarizes the current mechanisms and studies of ferroptosis in stroke-associated BMECs. In addition, we highlight the signaling pathways associated with ferroptosis in BMECs and current protocols for treating stroke by targeting and modulating ferroptosis in BMECs, which may provide innovative ideas for further research in stroke.

## 2 Concepts and functions of BMECS

Microvascular Endothelial Cells (MECs) are a single layer of flat epithelial cells that make up the inner wall of microvessels (<100 μm in diameter), which are distributed throughout the body in various tissues and organs, and are the key interface for the exchange of substances between the vascular system and the surrounding tissues (19). In the nervous system, BMVECs form the BBB through tight junctions (TJ) and adherens junctions (AJ), which are the first line of defense of the BBB, which selectively regulates the transport of substances between the blood and brain parenchyma, accurately controls intracerebral homeostasis, and plays a central role in regulating the blood-brain flux of endogenous and exogenous compounds and related metabolites (20). Once BMECs are damaged, the integrity of the BBB is compromised, which may further exacerbate brain edema, disrupt ionic homeostasis, alter signaling and immune infiltration, and lead to cell death (21). This mechanism is closely related to the pathophysiologic processes of many neurological disorders, including Stroke, Alzheimer’s disease (AD), Parkinson’s disease (PD), Multiple sclerosis (MS), and Traumatic brain injury (TBI) (22–25). Hence, the integrity of the BBB is critical to health. Previous studies on BMECs have focused on tightly linked structural complexes, angiogenesis and drug delivery (26–28), and studies related to the death mode of BMECs and the molecular mechanisms of subsequent BBB disruption are still to be refined.

## 3 Iron and ferroptosis

Iron is essential for cell survival and is also important for a variety of biological processes, such as DNA synthesis and oxygen transport, both of which require iron (29). The brain is a highly metabolically active and oxygen-consuming tissue that has a higher iron content than most organs (30). Iron is not only involved in the function of enzymes related to cellular energy metabolism, but also the synthesis of several neurotransmitters and the development of dendritic junctions are dependent on iron, thus iron is essential for normal brain function and activity (31). Of interest, as a transition metal, excess iron can catalyze the production of hydroxyl radicals via the Fenton reaction, accelerating the production of ROS and cellular lipid peroxidation, thereby inducing and exacerbating oxidative stress in neuronal cells (32). Thus, brain iron homeostasis is tightly regulated, and iron dysregulation can lead to severe pathological damage to the nervous system (33). Previous studies have also shown that poor clinical outcomes are strongly associated with elevated serum iron stores in stroke patients (34).

Ferroptosis is a non-apoptotic form of regulated cell death caused by iron-mediated accumulation of lipid peroxidation (35). Accumulation of iron-dependent lipid ROS is a typical feature of ferroptosis, and ROS lead to irreparable lipid damage and increased membrane permeability (36). Glutathione peroxidase 4 (GPX4) is recognized as an important biomarker of ferroptosis, regulating ferroptosis by reducing lipid peroxidation at the expense of glutathione(GSH) depletion (37). GPX4 converts GSH to oxidized glutathione (GSSG) and reduces cytotoxic lipid peroxide (L-OOH) to corresponding alcohol (L-OH), which ultimately reduces lipid ROS accumulation (38). Importantly, GPX4 dysfunction leads to uncontrolled oxidation of membrane polyunsaturated fatty acids (PUFA), resulting in massive ROS production and dysfunctional GSH production, which is a key pathological process in ferroptosis (39, 40). Undoubtedly, ferroptosis ultimately leads to the disruption of the integrity of the plasma membrane of cells or organelles (1), prompting the occurrence of deleterious pathological and physiological activities and secondary damage to the corresponding tissues. Thus, regulation of the balance between oxidative stress and the antioxidant system is an important molecular mechanism of ferroptosis (41).

## 4 Ferroptosis in BMECS and stroke

Ferroptosis has been shown to be involved in the pathological processes of several brain diseases, such as IS (42), CIRI (43), HS (17) and traumatic brain injury (44). Dietary iron supplementation enhances BBB reconstitution and protects neurons after stroke, study finds (45). Application of the 20 μM iron death inhibitor ferstatin-1(Fer-1) partially alleviated BBB disruption under hypoxia (46), suggesting that inhibition of ferroptosis may be a potential strategy for the treatment of a number of neurological disorders accompanied by BBB injury. Previous data have shown that BMECs, a key component of the BBB, control iron transport to the brain (47). This indicates that BMECs act as a gateway for iron transport through the BBB and may be associated with abnormal iron regulation and iron-dependent oxidative stress. Importantly, ferroptosis plays an important role in BBB dysfunction (48), and ferroptosis induces endothelial cell death (49). Whereas BMECs serve as a key component of the BBB, ferroptosis in BMECs is also important in disease development. Nevertheless, stroke-related iron death studies have focused on neurons and glial cells (42, 43, 50), and only a few studies (17, 18) have focused on the relationship between BMECs and ferroptosis after stroke. As such, ferroptosis in BMECs after stroke remains to be further investigated in depth.

### 4.1 Ferroptosis in BMECs and IS

IS (mainly occlusion of blood vessels supplying brain tissue) accounts for about 60 ~ 70% of all stroke cases (51), and its damage to the brain begins 20 minutes after stroke and can last for about 10 days (7). Common causes of IS are mainly embolization due to atherosclerosis of major carotid or intracranial arteries and cardiac dysfunction (51). The development of IS involves multiple physiological mechanisms, including cellular excitotoxicity, oxidative stress, neuroinflammation, and BBB destruction (52, 53). Among other things, BBB disruption is characterized by the rupture of TJs between BMECs, leading to increased permeability of the BBB as well as increased vesicular transport in the BMECs, allowing for the unregulated influx of blood-derived cells, macromolecules, and fluids, which ultimately leads to damaging cytotoxicity and vasculogenic edema, as well as life-threatening hemorrhagic transformations (21, 54). Moreover, the influx of iron into the brain following BBB disruption can trigger a series of interrelated metabolic responses, including ferroptosis, which plays a key role in the development of IS (55), and greatly impedes the recovery of neurological function after cerebral ischemia (56). Notably, patients with severe BBB injury had higher National Institutes of Health Stroke Scale scores, worse functional outcomes, and higher risk of death compared with patients with mild BBB injury (57, 58). Therefore, attenuating BBB damage improves the neurological prognosis of stroke patients (59).

**Figure 1:**
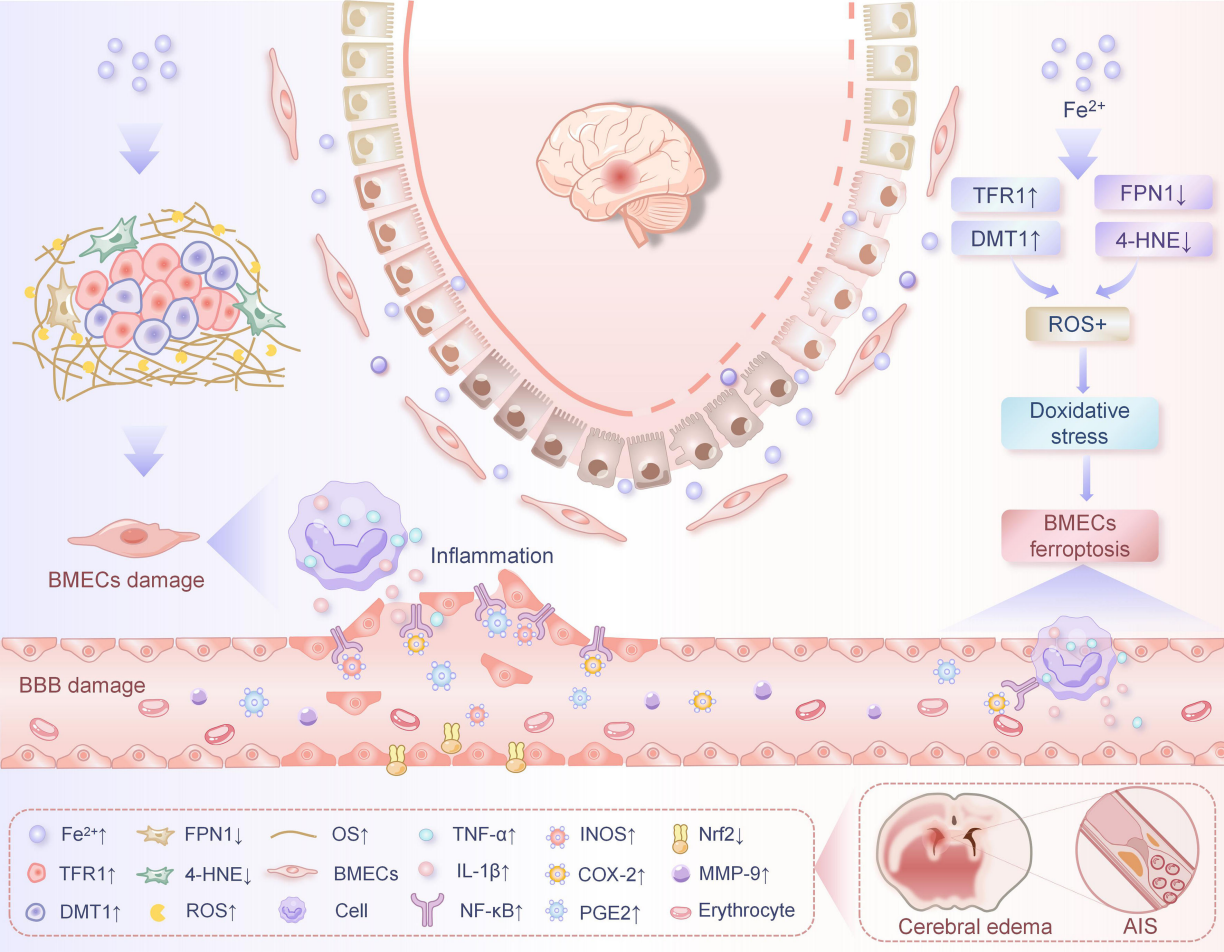
Ferroptosis in BMECs in relation to IS.

CIRI is an irreversible neurological impairment following reperfusion in IS patients. CIRI involves complex pathophysiological processes, including lipid peroxidation, glutamate release, and free radical generation (6). Among these mechanisms, ferroptosis has been demonstrated to play a pivotal role in CIRI (8). Moreover, accumulating evidence indicates that ferroptosis serves as a primary contributor to cell death associated with CIRI (60). Following CIRI, the dysfunction or death of BMECs disrupts TJs, directly compromising BBB integrity. This results in BBB leakage and cerebral damage, which represent early hallmarks of CIRI and act as triggering factors that exacerbate downstream inflammatory responses, apoptosis, and oxidative stress in neuronal cells (18). It is important that disruption of the BBB facilitates the extravasation of blood cells, macromolecules, and extracellular fluid into brain tissue, thereby increasing the risk of hemorrhage, inducing cerebral edema, and exacerbating inflammatory responses, which collectively promote the progression and expansion of injury (61, 62). Clinical studies indicate that the severity of BBB impairment correlates with reduced survival rates in IS patients (63). However, pharmacological agents that ameliorate BBB injury have been demonstrated to effectively improve outcomes in both IS patients and stroke model mice (59, 64). Consequently, it is imperative to investigate the mechanisms underlying ferroptosis in BMECs following IS (**Figure 1**), with the ultimate goal of preserving BBB integrity and enhancing clinical prognosis.

In studying the role and mechanism of mitochondrial ferritin (FtMt) and BBB destruction during cerebral ischemia-reperfusion, Wang et al. (18) found that iron-mediated oxidative stress in BMECs is an early cause of BMECs injury and BBB destruction in IS. They found that FtMt was significantly upregulated in BMECs on the affected side following CIRI. FtMt overexpression was demonstrated to protect BBB integrity by enhancing endothelial TJs expression, thereby reducing vascular leakage post-CIRI. Mechanistically, their findings revealed that CIRI induces iron dysregulation and accumulation in BMECs. FtMt was shown to prevent CIRI-induced upregulation of iron uptake proteins (transferrin receptor 1 [TfR1] and divalent metal transporter 1 [DMT1]) in BMECs, while attenuating the decrease of ferroportin 1 (FPN1, the sole cellular iron exporter) and 4-hydroxynonenal (4-HNE, a lipid peroxidation end product and biomarker of oxidative stress) levels in BMECs. This mechanism further blocked ROS generation and accumulation, consequently suppressing BMECs ferroptosis and BBB disruption. These results indicate that modulating iron metabolism-related proteins to inhibit iron overload in BMECs may mitigate CIRI-induced BBB damage. The aforementioned findings may provide novel insights for treatment plans of IS.

### 4.2 Ferroptosis in BMECs and HS

HS refers to intracranial hemorrhage caused by increased vascular fragility and subsequent rupture of blood vessels within the brain parenchyma in the absence of trauma (65). In the United States, Europe, and Australia, HS accounts for approximately 10-15% of all stroke cases, whereas in Asia, its ranges from 20-30% (66). During HS, vascular rupture occurs, allowing blood to extravasate through the disrupted BBB into brain tissue or ventricular spaces, thereby elevating intracranial pressure and reducing cerebral blood flow (67, 68), which severely impairs neurological function. Following HS onset, blood components initiate pathological processes including cellular excitotoxicity, cytotoxic edema, oxidative stress, and neuroinflammation, which further compromise BBB integrity. These neurotoxic blood-derived factors comprise thrombin, fibrin, and erythrocyte components (62, 69). Thrombin, a cascade product of prothrombin during post-hemorrhagic hemostasis, induces subsequent BBB disruption by binding to protease-activated receptor 1 (PAR-1), which triggers Src kinase phosphorylation and microglial activation (70). Iron degradation from hemoglobin is one of the most important factors in HS-induced BBB hyperpermeability (71).

Secondary injury following HS is a destructive consequence mediated by Fe^2+^-catalyzed oxidative reactions (72, 73). Studies have demonstrated that hemoglobin-induced neuronal death and iron deposition in organotypic hippocampal slice cultures and primary cortical neurons can be attenuated by the administration of Fer-1 or other ferroptosis inhibitors (74, 75). Fer-1 prevents hemoglobin-induced GPX4 deficiency and lipid ROS accumulation, while mitigating tissue damage and neurological deficits in mice following HS (74). Moreover, cyclooxygenase 2 (COX-2), an enzyme encoded by the PTGS-2 gene, was significantly upregulated in the first three days after ICH in mice. Notably, this increase in COX-2 could be suppressed by Fer-1 (74). The study also demonstrated that N-acetylcysteine (NAC) could attenuate hemin-/hemoglobin-induced primary cortical neuronal cell death and improve functional recovery in mice following HS (76). Furthermore, selenium was found to enhance the transcriptional response of GPX4 by coordinately activating transcription factors TFAP2c and Specific protein 1 (SP1), thereby protecting neurons from ferroptosis and improving neurological function in post-HS mice (37). These findings suggest a close association between HS and ferroptosis.

**Figure 2:**
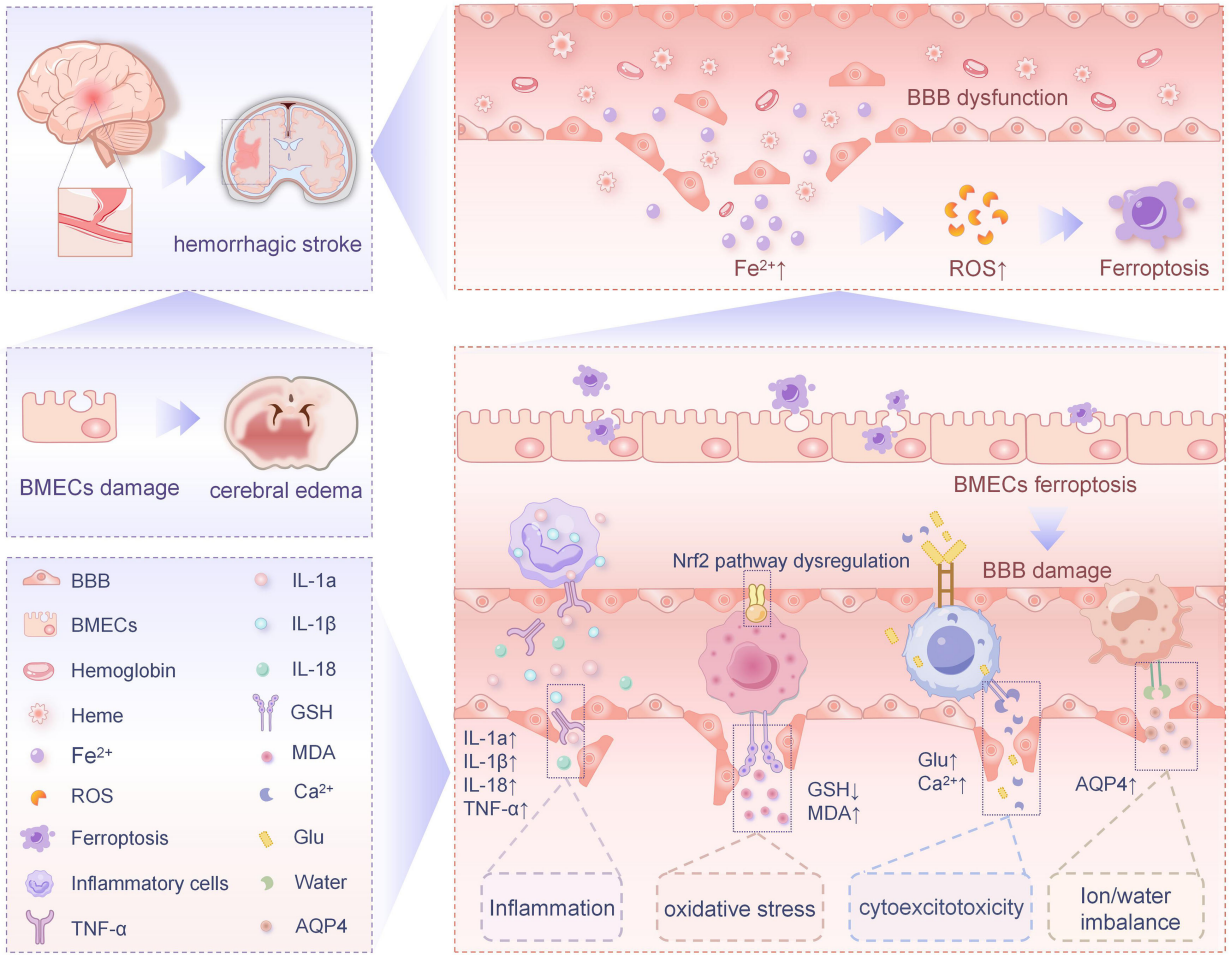
Ferroptosis in BMECs in relation to HS.

Both *in vivo* and ex vivo experimental models have demonstrated that hemoglobin (Hb) and hemin exert cytotoxic effects on neurons through inflammatory responses (77, 78). During the early stage of HS, Hb and hemin do not induce immediate cellular damage; however, the accumulation of Fe^2+^ derived from Hb/hemin conversion (or release) can be observed in BMECs, which may ultimately lead to cytotoxicity. In the context of BBB dysfunction, intracellular Fe^2+^ within BBB components (including BMECs) has been demonstrated to contribute to subsequent brain injury following HS (79). This suggests that Fe^2+^ accumulation in BMECs and pericytes induces BBB impairment during secondary brain injury post-HS, while modulation of iron metabolism in BMECs may potentially mitigate HS-induced BBB disruption (**Figure 2**). These findings provide novel insights for developing treatment strategies for HS.

## 5 Relevant signaling pathways regulating ferroptosis in BMECS

Iron has been demonstrated to mediate BBB dysfunction primarily through the generation of ROS, which can directly induce endothelial cell degradation and activate related signaling pathways (80). Cytokine-mediated signaling pathways also constitute a critical component of the inflammatory process following stroke. For instance, both tumor necrosis factor-alpha (TNF-α) and interleukin-1 beta (IL-1β) have been shown to mediate cellular functions, including BMECs death, subsequently leading to increased BBB permeability (81–83). In summary, researchers have devoted increasing efforts to investigating relevant pathways to identify potential neuroprotective targets for stroke treatment. Below is our summary of the signaling pathways regulating ferroptosis in BMECs (**Table 1**). We anticipate that novel drugs targeting the modulation of BMECs ferroptosis could be developed based on these pathways for stroke prevention and treatment. Furthermore, we aim to explore additional related pathways based on existing findings to provide a broader array of therapeutic targets for mitigating BMECs ferroptosis.

**Table 1:** Specific mechanisms regulating ferroptosis in BMECs related signaling pathways.

Models	Animals/Cells	Adjustment method	Signaling pathway	Primary endings	Ref.
OGD+HGR	SD rats and RBMVECs	Inhibition	Meg3/p53/GPX4	Inhibition of ferroptosis in BMECs	(84)
OGD/R and MCAO/R	SD rats and HBMVECs	Inhibition	TEAD1/MMP3		(85)
CIRI and OGD/R	C57BL/6 mice and bEND.3 cells	Activation	SESN2/SystemX_c_^−^ /GPX4		(86)
MCAO/R and OGD/R	SD rats and HBEC-5i	Inhibition	SP1/TNFSF9/SLC3A2		(87)

### 5.1 Meg3/p53/GPX4 signaling pathway

As a class of non-coding RNAs with lengths exceeding 200 nucleotides, long non-coding RNAs (lncRNAs) participate in numerous profound physiological and pathophysiological processes—including cell differentiation, proliferation, immunity, inflammation, and apoptosis—through the regulation of their target genes (88, 89). Previous experimental evidence has demonstrated that dysregulation of lncRNAs is closely associated with neurological dysfunction following stroke (90–92). Maternally expressed gene 3 (Meg3) is a newly identified lncRNA located on human chromosome 14q32, exhibiting strong genetic susceptibility in neurovascular and neurodegenerative diseases (93). Accumulating evidence indicates that “upregulation of Meg3 expression” is positively correlated with poor prognosis in IS, whereas “inhibition of Meg3 expression” *in vivo* and ex vivo exerts potent neuroprotective effects against CIRI (94, 95). The tumor suppressor p53 has been identified as a downstream target of Meg3 involved in initiating cellular responses to endogenous or exogenous stress (96). and p53 plays an important role in mediating the cellular response to oxidative stress-induced ferroptosis (97). Previous studies have also confirmed that Meg3 can contribute to p53-mediated ischemic neuronal death (98). Therefore, it is mechanistically plausible that the Meg3/p53 signaling pathway regulates IS through mediating cellular ferroptosis.

**Figure 3:**
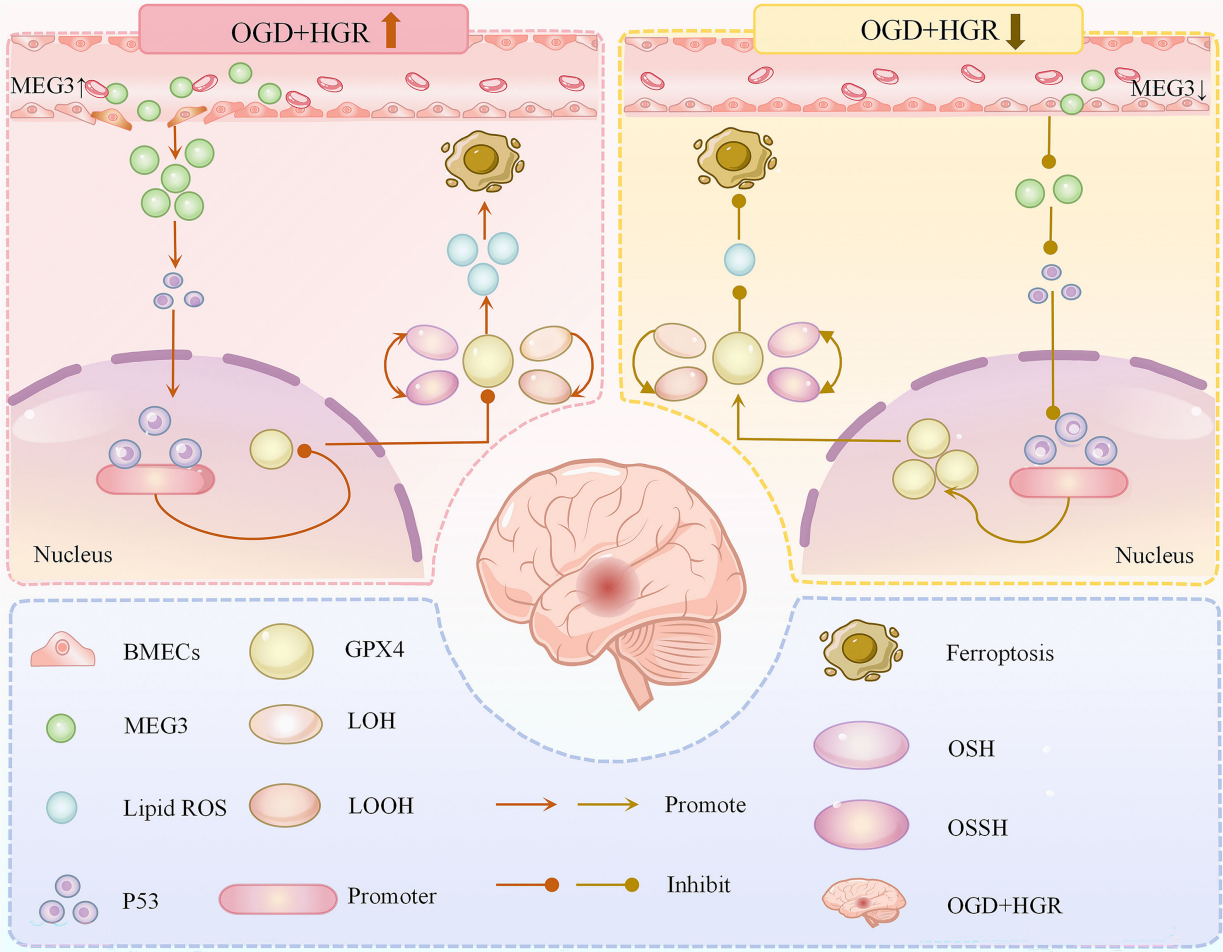
Relationship between Meg3/p53/GPX4 signaling pathway and ferroptosis in BMECs.

Chen et al. (84) simulated *in vitro* diabetic cerebral ischemic injury by incubating rat BMECs with different concentrations of glucose for 24 h after 6 h exposure to oxygen and glucose deprivation (OGD) conditions. The results revealed that in BMECs exposed to OGD combined with hyperglycemic reperfusion (HGR), the level of Acyl-CoA synthetase long-chain family member 4 (ACSL4) (a positive regulator of ferroptosis) levels were significantly increased, and intracellular iron concentration, lipid ROS production, and lipid peroxidation product MPO were elevated, whereas the levels of two negative regulators of ferroptosis, GPX4 and ferritin heavy chain 1 (FTH1), were decreased, and GSH levels and GSH/GSSG ratios were also significantly reduced. Interestingly, by silencing Meg3 not only significantly inhibited the expression of ACSL4 and the lipid peroxidation product MPO, decreased intracellular iron concentration, and reduced lipid ROS generation, but also reversed the levels of FTH 1 and GPX4 and the ratio of GSH/GSSG. Concurrently, this study demonstrated that Meg3 knockdown markedly inhibited the OGD+HGR injury-induced elevation of p53 gene and protein expression, confirming p53 as a downstream target of Meg3. To further investigate the biological function of p53 in OGD+HGR-induced injury, they evaluated the effect of p53 on OGD+HGR-induced ferroptosis. It was found that inhibition of p53 significantly increased cell survival, resulting in increased cell viability and decreased LDH release. Knockdown of the p53 gene not only decreased cellular iron concentration, lipid ROS production and MPO levels, but also increased GSH content and GSH/GSSG ratio, while overexpression of p53 had the opposite effect. In addition, they found that GPX4 protein expression is significantly downregulated by p53 overexpression and upregulated by p53 deletion in OGD+HGR-injured BMVECs. They further found that p53 structurally binds to the GPX4 promoter by chromatin anti-p53 immunoprecipitation experiments, and that p53 interacts with the GPX4 promoter to repress its transcription in the presence of OGD+HGR. Collectively, these data indicate that OGD+HGR injury triggers hallmark events of ferroptosis in BMECs accompanied by elevated Meg3 expression. Inhibition of the Meg3/p53 signaling pathway confers protection against OGD+HGR-induced ferroptosis in BMECs by modulating GPX4 transcription and expression (**Figure 3**).

### 5.2 TEAD1/MMP3 signaling pathway

TEA structural domain transcription factor 1 (TEAD1), a member of the TEAD family, plays an important role in the regulation of cellular activity, tissue regeneration, and stem cell maintenance (99). The BBB is primarily modulated through interactions between its intracellular components and the extracellular matrix (ECM), as the principal driver of ECM degradation and remodeling, matrix metalloproteinases (MMPs) are critically involved in BBB disruption (100). Matrix metalloproteinase-3 (MMP3), a member of the MMP family, is a zinc-dependent protease activated by self-cleavage, and activated MMP3 remodels the basement membrane of the ECM, thereby protecting the BBB (101).

**Figure 4:**
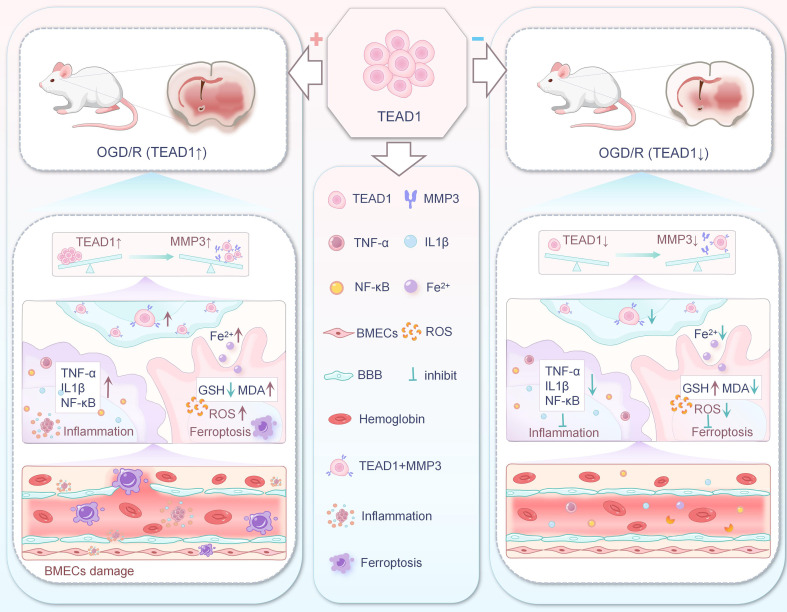
Relationship between TEAD1/MMP3 signaling pathway and ferroptosis in BMECs.

Lu et al. (85) conducted *in vivo* and *in vitro* experiments in order to explore the molecular mechanisms associated with TEAD1 and MMP3 in CIRI. In ex vivo experiments, they established an I/R model using oxygen-glucose deprivation/reoxygenation (OGD/R) of BMECs, detected the mRNA and protein expression of TEAD1 and MMP3 by RT-qPCR and protein blotting, assessed ferroptosis using a kit, and RNA immunoprecipitation assay demonstrated the interaction between TEAD1 and MMP3. In *in vivo* experiments, they induced CIRI in rats by the Middle cerebral artery occlusion/reperfusion (MCAO/R) model using tetrazolium chloride staining, EVANS blue extravasation, neurological function scores, and cerebral water content assays Brain damage in rats was assessed. It was found that OGD/R significantly upregulated MMP3 expression in BMECs, and the knockdown of MMP3 alleviated both apoptosis and ferroptosis in OGD/R-treated BMECs. TEAD1 enhanced MMP3 expression by targeting its promoter, whereas silencing TEAD1 reduced MMP3 levels, thereby attenuating OGD/R-mediated inflammation and ferroptosis. In the MCAO/R model, TEAD1 inhibition also significantly modulated MMP3 expression, protecting rat brain tissues from CIRI. These findings indicate that TEAD1 promotes cerebral inflammation and ferroptosis in both ex vivo and *in vivo* I/R models by transcriptionally regulating MMP3, suggesting that suppression of the TEAD1/MMP3 signaling pathway mitigates ferroptosis in BMECs and ameliorates CIRI (**Figure 4**).

### 5.3 SESN2/System X_c_^−^/GPX4 signaling pathway

SESN2 is a highly conserved stress-inducible protein. A variety of stressors, such as hypoxia, oxidative stress, and inflammation, can upregulate SESN2 expression (102, 103). As a key antioxidant regulator, SESN2 can alleviate the stress response by reducing ROS levels and inhibiting mammalian target of rapamycin complex 1 (mTORC1) (103). Previous studies have shown that induction of SESN2 effectively ameliorates ferroptosis (6), and is protective against CIRI (104).

**Figure 5:**
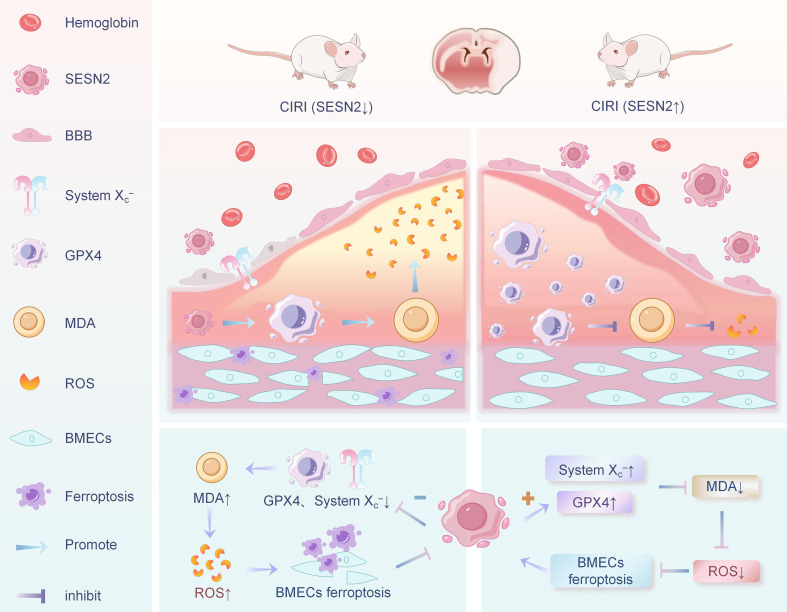
Relationship between SESN2/System X_c_^-^/GPX4 signaling pathway and ferroptosis in BMECs.

The System X_c_^-^/GSH/GPX4 signaling pathway is a typical pathway that monitors ferroptosis, and blockage of this pathway induces cellular sensitivity to ferroptosis (1). In a focal CIRI model established in C57BL/6 mice, Hu et al. (86) observed elevated indicators of ferroptosis in BMECs post-CIRI, accompanied by reduced expression of ferroptosis-related proteins (System X_c_^−^ and GPX4). Subsequent RNA sequencing and bioinformatics analyses systematically examined the enrichment and interaction networks of differentially expressed genes in OGD/R-induced bEND.3 cells, identifying six ferroptosis-associated hub genes. SESN2 was determined to be both a key ferroptosis-related hub gene and a critical antioxidant regulator. Silencing SESN2 downregulated the expression of System X_c_^−^ and GPX4, whereas SESN2 overexpression promoted their expression. These findings demonstrate that SESN2 serves as a negative regulator of ferroptosis. Therefore, upregulation of SESN2 expression and activation of the System X_c_^−^/GPX4 pathway can inhibit ferroptosis in BMECs. Based on RNA sequencing and bioinformatics analysis, this study untangles the pivotal role of ferroptosis in CIRI-induced BMEC injury, demonstrating that the SESN2/System X_c_^−^/GPX4 pathway exerts negative regulatory effects on CIRI-induced ferroptosis in BMECs (**Figure 5**). These findings will facilitate future mechanistic investigations of CIRI-induced ferroptosis in BMECs and provide a theoretical foundation for developing therapeutic strategies and targeted drugs against CIRI.

### 5.4 SP1/TNFSF9/SLC3A2 signaling pathway

Specificity protein 1 (SP1), a transcription factor, targets GC-rich sites in the promoter regions of numerous genes to upregulate their transcriptional output (105). As reported by Pei et al. (106) in a short-term high-fat diet rat model, SP1-induced ACSL4 overexpression mediated FUNDC1 deficiency, thereby promoting cardiac remodeling and contractile dysfunction through triggering ferroptosis in cardiac cells. Tumor necrosis factor superfamily member 9 (TNFSF9), a member of the TNF superfamily. TNFSF9 and its receptor TNFRSF9 has been shown to play an important role in the interaction between antigen-presenting cells (APCs) and T cells or Natural killer (NK) cells enhance cellular activation and prolongs survival (107, 108). Activation of TNFSF9 signaling in epithelial and endothelial cells has been reported to induce sterile inflammatory responses by recruiting immune cells (108, 109). Furthermore, Li et al. (110) demonstrated that TNFSF9 accelerated microglia activation and ferroptosis in the MCAO/R mouse model. Fann et al. (111) also demonstrated that disruption of TNFSF9-TNFRSF9 interactions ameliorated inflammation-associated brain tissue injury following cerebral ischemia-reperfusion. Solute carrier family 3 member 2 (SLC3A2) is one of the two subunits of the System X_c_^−^ cysteine/glutamate antiporter (a transmembrane protein complex consisting of the SLC7A11 and SLC3A2 subunits) (1). This delivery system is essential for the synthesis of reduced glutathione (GSH) and GPX4, as well as the clearance of ROS during oxidative stress, and plays a key role in the execution of the System X_c_^−^/GSH/GPX4 pathway to monitor ferroptosis (1). Importantly, it has been found that chlorogenic acid ameliorates hypoxic-ischemic brain damage in neonatal mice by inhibiting the expression of the aforementioned ferroptosis-related factors (GPX4, SLC7A11 and SLC3A2 protein complex) (112).

**Figure 6:**
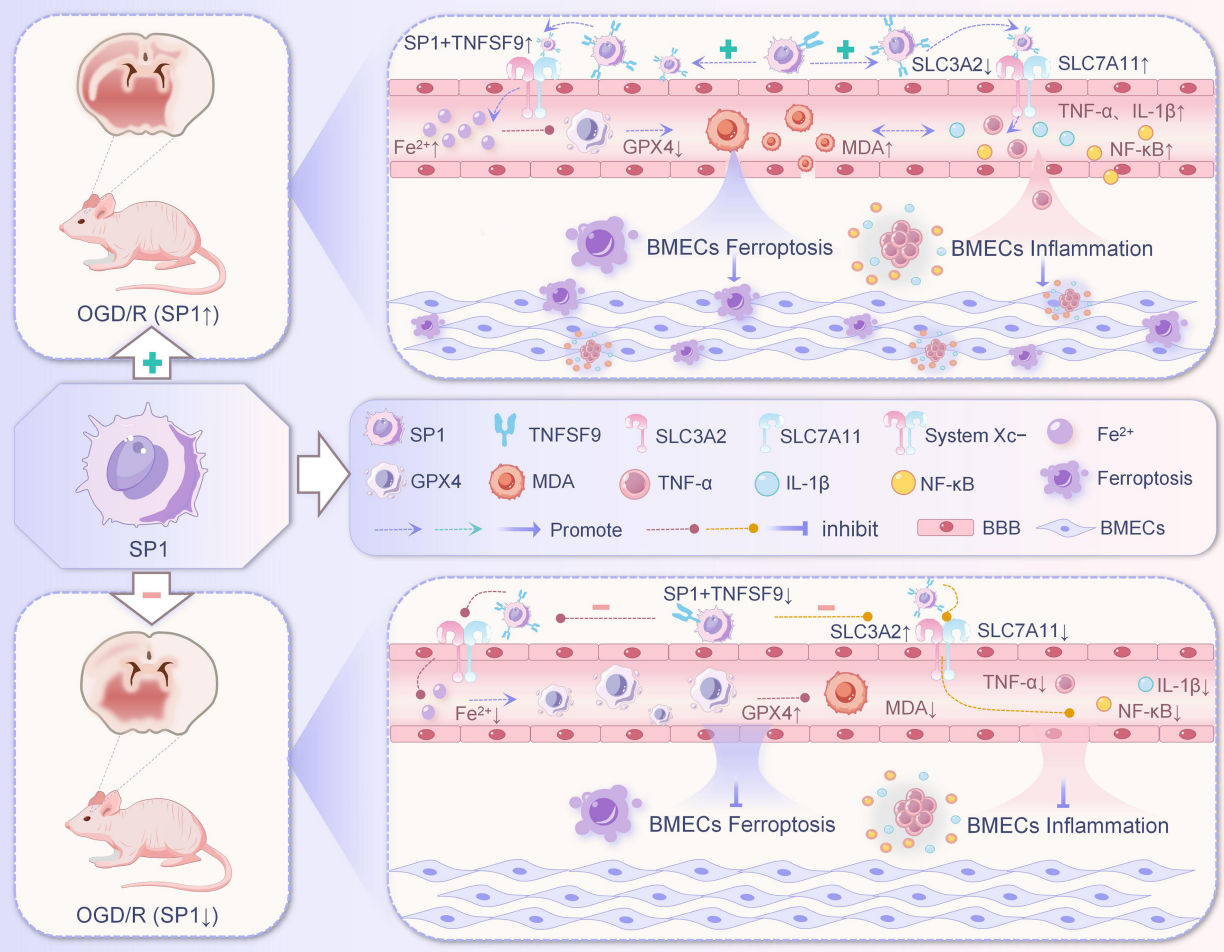
Relationship between SP1/TNFSF9/SLC3A2 signaling pathway and ferroptosis in BMECs.

Endothelial cells also play an essential function during ischemia-reperfusion injury, and their oxidative stress can lead to an inflammatory response in injured tissues through the recruitment of neutrophils and their prothrombotic effects (105, 113). Liang et al. (87) demonstrated elevated plasma TNFSF9 mRNA levels in CIRI patients, while TNFSF9 silencing suppressed ferroptosis, apoptosis, and inflammatory mediator release in BMECs subjected to OGD/R treatment. As one of the transcriptional factors of TNFSF9, SP1 exhibited a positive regulatory role in TNFSF9 expression. TNFSF9 overexpression reversed the inhibitory effects of SP1 silencing on ferroptosis, apoptosis, and inflammatory mediator release in OGD/R-treated BMECs. Ex vivo experiments revealed that SLC3A2 silencing abolished the protective effects of TNFSF9 downregulation in BMECs under OGD/R conditions. *In vivo* studies demonstrated that TNFSF9 silencing attenuated CIRI-induced cerebral infarct volume in rats by modulating SLC3A2 expression. Collectively, these findings indicate that TNFSF9, regulated by SP1, exacerbates ferroptosis, apoptosis, and inflammatory mediator release in BMECs under both ex vivo and *in vivo* OGD/R conditions by suppressing SLC3A2 expression, thereby aggravating CIRI. In conclusion, this study reveals that SP1-regulated TNFSF9 promotes ferroptosis, apoptosis, and inflammatory mediator release in BMECs under OGD/R conditions while increasing cerebral infarct volume in rats through SLC3A2 regulation (**Figure 6**). These results suggest that the SP1/TNFSF9/SLC3A2 axis constitutes a critical signaling pathway modulating ferroptosis in BMECs and may serve as a potential therapeutic target for stroke treatment.

## 6 Stroke therapeutic strategies for targeting and modulating ferroptosis in BMECS

### 6.1 Inhibitors of ferroptosis

Iron inhibitors, iron chelators (e.g., ferrostatin-1), and deferoxamine (DFX) exert significant protective effects in stroke mice (33). Abdul et al. (114) investigated the impact of iron chelation with DFX on cerebrovascular patterns and functional outcomes in diabetic rats following stroke. The results demonstrated that DFX treatment (100 mg/kg, i.p.) prevented vascular degeneration and microglial activation in diabetic rats, while improving AQP4 polarity and BBB permeability. Furthermore, iron markedly increased markers of ferroptosis and lipid ROS in BMECs of diabetic animals; however, DFX effectively attenuated these effects. These findings suggest that DFX represents a critical strategy for inhibiting ferroptosis in BMECs.

Li et al. (115) conducted a comparative study between diabetic female and male rats to investigate hemin-induced ferroptosis in BMECs. The results demonstrated that hemin mediated ferroptosis in BMECs of both sexes, whereas DFX(100 mg/kg,i.p., Sigma-Aldrich) prevented hemin-induced BMECs ferroptosis in diabetic rats. DFX treatment attenuated diabetes-mediated gliovascular remodeling and BBB disruption while improving memory function and survival rates in diabetic subjects. These findings propose a novel therapeutic strategy for preventing post-stroke ferroptosis in BMECs.

### 6.2 Genes and molecules

#### 6.2.1 Fibroblast growth factor 2

Endothelial cell death is a common feature of ischemia/reperfusion (I/R) injury (116), and microvascular injury is a determinant of I/R injury in all tissues (117). Therefore, attenuating microvascular endothelial cell death is a key strategy for treating I/R injury. In the fibroblast growth factor (FGF) family, FGF2 is an important component, and FGF2 regulates a variety of cellular processes through binding and activation of FGF receptors (118). Previous studies have shown that FGF2 promotes angiogenesis, wound healing, and tissue regeneration (49, 119), minimizes infarct size and improves microvascular dysfunction (120, 121). NFELE2 is a transcription factor considered to be a major defense mechanism against oxidative stress that coordinates the activation of cytoprotective genes (122). However, FGF2 enhances NFELE2 expression and attenuates I/R injury (123, 124). A study demonstrated that FGF2 exhibits potent antioxidant properties, effectively suppressing ferroptosis levels to an extent comparable with ferrostatin-1 (Fer-1, a ferroptosis inhibitor) treatment (125). Furthermore, FGF2 was shown to mitigate endothelial cell death in I/R injury by inhibiting ferroptosis. Notably, the previously suppressed FGF2-induced endothelial cell death was significantly augmented following treatment with erastin (a ferroptosis inducer), indicating that erastin can markedly reverse the protective effects of FGF2 against ferroptosis. These findings substantiate that ferroptosis participates in I/R injury, and that FGF2 confers protection against I/R injury by attenuating ferroptosis in endothelial cells.

#### 6.2.2 p23

p23 is a protein with a molecular weight of 23 kDa that is widely expressed in tissues such as heart, brain, kidney, and lung (126). Structural studies reported that the N-terminal end of the p23 protein contains a β-sheet structural domain consisting of two inverted β-sheets, and its C-terminal end is an unfolded tail (127, 128). Importantly, P23 often serves unique biological functions as a molecular chaperone and regulates its biological functions by binding to various proteins that play regulatory roles in a wide range of diseases. First, it is an important co-molecular chaperone of HSP90, assisting HSP90 in its molecular chaperone function through its C-terminal co-molecular chaperone complexes (129). However, p23 has an HSP90-independent role in intracellular receptor-regulated gene expression and displays features of ribosome biogenesis and vesicle-mediated translocation (130). p23 was and is a co-chaperone of ferroptosis and is also known as cytosolic prostaglandin E (PGE) synthase (126). The biochemical mechanisms of ferroptosis mainly involve iron-dependent lipid ROS formation, GSH depletion or GPX4 inactivation (131, 132). And the COX-2/PGE2 pathway has been reported to be closely associated with CIRI and ferroptosis (133, 134). Recent studies have also shown that PGE2 can effectively prevent CIRI-induced ferroptosis (45, 135).

Zhao et al. (136) examined the effects of p23 on CIRI-induced BBB dysfunction and ferroptosis in BMECs and its mechanism in order to clarify the effects of CIRI or OGD/R on ferroptosis in BBB and BMECs as well as on p23 expression. The results showed that the permeability of BMECs increased after the occurrence of CIRI or OGD/R. Importantly, ferroptosis was participated in OGD/R-induced injury to BMECs, and inhibition of ferroptosis in BMECs had a protective effect on CIRI-induced BBB injury. They found that p23 was significantly upregulated in both *in vitro* and *in vivo* models. Moreover, molecular docking and co-immunoprecipitation experiments showed that p23 also formed a complex with HSP90 and GPX4, a major regulator of ferroptosis, through its N-terminal structure (1-90aa), which improved their stability, inhibited the degradation of GPX4, and ultimately suppressed ferroptosis in BMECs, thereby protecting against CIRI-induced BBB injury. In conclusion, this study demonstrates that p23, a key regulator of BBB injury after CIRI, can attenuate stroke-induced BBB injury by enhancing GPX4 stability and inhibiting ferroptosis in BMECs. This provides a potential therapeutic target for stroke.

#### 6.2.3 Methyltransferase-like 3

N6-methyladenosine (m6A) is the most common modification in epigenetic changes that drive a variety of biological functions, including promotion of tissue development, stem cell differentiation and repair, protein translation, RNA splicing, and DNA damage response (137, 138). Methyltransferase -like 3 (METTL3) is a member of the m6A methyltransferase complex that catalyzes the modification of the m6A (139). Recently, Wang et al. (140) demonstrated that METTL3-mediated m6A modification is closely associated with mammalian cerebellar development. Huang et al. (141) also showed that the abnormal expression and distribution of METTL3 in the hippocampal region of the Alzheimer’s disease brain may underlie the regulation of changes in the expression of genes associated with the pathogenesis of the disease. These studies suggest that METTL3 is involved in brain development.

Zhang et al. (142) in order to explore whether METTL3 could alleviate ICH by blocking iron death, used OGD and hemagglutinin to stimulate BMVECs to establish an ICH model. Cell viability was detected using CCK8 assay. The levels of Fe^2+^, GSH, ROS, LPO and MDA were detected using the corresponding kits. Relative protein expression was detected using Western blotting and RT-qPCR. Cell death was analyzed using TUNEL and propidium iodide staining. The correlation between METTL3 and GPX4 was analyzed by Spearman correlation test and further confirmed by CHIP method. The results demonstrated that in both oxygen and glucose deprivation hemin (OGD/h) -treated BMECs and ICH mice, elevated levels of Fe^2+^, ROS, LPO, and MDA were observed, accompanied by reduced GSH. Furthermore, OGD/h-treated BMECs exhibited decreased cell viability and SLC7A11 protein expression, along with increased cell death and TFR1 protein levels. Notably, METTL3 silencing alleviated OGD/h-induced BMVEC injury. In addition to this, GPX4 is a key protein in the inhibition of lipid peroxidation, and a large body of evidence suggests that GPX4 serves as a reference marker for determining ferroptosis (143). Inactivation of GPX4 leads to a disruption of oxidative homeostasis and disruption of membrane structure by lipid peroxidation, which activates ferroptosis (144, 145). It is worth noting that, this study also demonstrated a significant negative correlation between METTL3 and GPX4, which was further confirmed by CHIP assays (142). Silencing of METTL3 reduced m6A modification of GPX4 while increasing GPX4 mRNA levels. Knockdown of GPX4 was found to neutralize the effects of METTL3 in OGD/h-treated BMECs. These results collectively indicate that ferroptosis occurs in both OGD/h-treated BMECs and the ICH mouse model. Importantly, METTL3 silencing effectively inhibited ferroptosis through regulation of m6A modification and GPX4 mRNA levels. These findings provide novel insights into the inhibition of ferroptosis in BMECs for stroke treatment.

#### 6.2.4 Long non-coding RNA H19

Long-noncoding RNAs (lncRNAs) are members of the non-coding RNA family, with lengths ranging from 100 to 200 nt, and regulate a variety of diseases through competitive binding with micrornas (miRNAs) (146, 147), miRNAs further target downstream genes (148). Among them, LncRNA H19 plays an essential role in angiogenesis, adipocyte differentiation, lipid metabolism, inflammatory response, cell proliferation and apoptosis (149–152). Kim et al. (153) found that the expression of lncRNA H19 was significantly upregulated in an ICH model. Therefore, LncRNA H19 may be a biomarker for ICH. Importantly, Bai et al. (154) showed that LncRNA H19 may influence ferroptosis. Previous studies have found that miR-106b-5p is considered a diagnostic biomarker for acute stroke and ischemic events (155, 156). Besides, ACSL4 was identified as an important regulator of ferroptosis (157). Hence, miR-106b-5p and ACSL4 are closely associated with stroke-related ferroptosis.

Chen et al. (65) explored the role of lncRNA H19 in ICH and its potential molecular mechanisms. They used real-time quantitative polymerase chain reaction (RT-qPCR) to detect mRNA expression. Cell viability was analyzed using Cell Counting Kit 8 (CCK8). Ferroptosis in BMECs was detected using PI-stained flow cytometry and. Targeting relationships were predicted using Starbase and TargetScan and verified by RNA pull-down and luciferase reporter gene assays. Protein expression was detected using Western blotting. It was found that LncRNA H19 was highly expressed in ICH model cells. Overexpression of LncRNA H19 was observed to inhibit cell viability and promote ferroptosis in bone marrow vascular endothelial cells. In this study, miR-106b-5p was predicted as a target miRNA of LncRNA H19. Notably, miR-106b-5p expression was significantly downregulated in OGD/R-treated cells. Overexpression of miR-106b-5p could reverse the effects of LncRNA H19 on both cell viability and ferroptosis in BMECs. Furthermore, ACSL4 was highly expressed in OGD/R-treated cells. Upregulation of ACSL4 was demonstrated to counteract the effects of miR-106b-5p and induce BMECs dysfunction. In conclusion, LncRNA H19 is overexpressed in ICH, and its knockdown may promote cell proliferation while inhibiting ferroptosis in BMECs through regulation of the miR-106b-5p/ACSL4 axis. Therefore, LncRNA H19 knockout represents a potentially promising therapeutic strategy for stroke treatment.

### 6.3 Chemical compound

#### 6.3.1 Rosmarinic acid liposomes

Rosmarinic acid (RosA) is an abundant herbal monomer in Salvia miltiorrhiza or Perilla leaf with anti-inflammatory, antioxidant, antitumor and antiplatelet aggregation properties (158). RosA is also used in the treatment of IS, HS (159), and other CNS disorders (160–162). Previous studies have demonstrated that RosA is able to selectively chelate ferric ions by acting as an iron chelator (163). RosA is a naturally occurring water-soluble phenolic acid compound characterized by its unstable nature, low lipid solubility and limited cell membrane permeability (158, 164). However, most CNS drugs have limited penetration into the brain, mainly due to the presence of BBB. Therefore, nanomaterials with affinity for BBB have emerged as promising research directions for the treatment of CNS disorders, which offer enhanced drug loading capacity, controlled release kinetics, improved stability, biocompatibility and reduced toxicity (165, 166).

Transferrin receptor 1 (TfR1) is present on the surface of many types of cells, and iron uptake in the brain is primarily facilitated by the transferrin-transferrin receptor protein 1 (Tf-TfR1) system facilitated (167), and TfR1 transports Fe^3+^ carried by transferrin to the cytoplasm by binding to transferrin, which is the main mechanism of iron uptake in cells (168). Furthermore, TfR1 has been shown to promote cellular ferroptosis (168, 169). Therefore, regulating TfR1 expression in BMECs may be a potential pathway for stroke intervention. Jia et al. (170) used Wild-type (WT) and TfR1^EC^ cKO (specific knockout of TfR1 gene in BMECs) mice to establish the distal middle cerebral artery occlusion (dMCAO) model. The potential neuroprotective effect of nanoliposome-encapsulated Rosmarinic acid liposomes (RosA-LIP) on ischemic stroke was explored by simultaneously administering RosA-LIP (20 mg/kg/d, i.p.) treatment. It was found that RosA-LIP could inhibit ferroptosis by ameliorating abnormal mitochondrial function, increasing GPX4 levels, and decreasing ACSL4/LPCAT3/Lox-dependent lipid peroxidation. Moreover, RosA-LIP effectively improved BBB permeability, increased TJs protein expression, and reduced iron levels in ischemic tissues and BMECs by down-regulating the expression of the iron storage proteins FTL and FTH, up-regulating the expression of the iron export protein FPN1, and down-regulating the expression of the iron uptake proteins DMT1(-ire), DMT1(+IRE), and TFR1. Significantly, in dMCAO-treated TfR1^EC^cKO mice, RosA-LIP was able to attenuate ACSL4/LPCAT3/Lox-mediated ferroptosis through inhibition of TfR1, improve its stability and precise delivery in serum and brain, and effectively alleviate ischemia-induced behavioral abnormalities and pathological damage. In conclusion, this study confirms that RosA-LIP can effectively increase RosA levels in the brain by regulating TfR1 in BMECs, thus exerting a neuroprotective effect of inhibiting ferroptosis.

#### 6.3.2 Se-methyl L-selenocysteine

Selenium (Se) is a trace element that is essential for a variety of metabolic processes, including protection against oxidative stress and maintenance of normal cardiovascular function (171, 172). To date, 25 genes encoding selenoproteins have been identified in humans and 24 in rodents, of which glutathione peroxidases (GPXs) are involved in antioxidant action, while selenoprotein P is a plasma and extracellular protein that distributes selenium throughout the body via the circulatory system and helps maintain selenium homeostasis (173–175). The protective effect of selenium against stroke is partially understood (176, 177), and several clinical studies have shown that circulating selenium concentrations, as well as dietary selenium, are negatively correlated with the incidence of stroke, suggesting that selenium supplementation may be beneficial in acute ischemic stroke (178).

Se-methyl L-selenocysteine (SeMC) is a new Se supplement characterized as the methyl derivative of selenocysteine (the 21st indispensable amino acid). Fei et al. (179) elucidated the protective mechanism of SeMC from the perspective of BBB function maintenance. The researchers established an *in vivo* model by subjecting C57BL/6J mice to MCAO/R treatment. Concurrently, an ex vivo model was developed by exposing bEnd.3 cells (mice BMECs) to OGD/R conditions. They found that in the MCAO/R model, GPX4 expression was downregulated, while ACSL4 expression was upregulated, indicating that CIRI triggered significant ferroptosis. Notably, treatment with SeMC markedly reversed this process. Furthermore, the levels of 4-HNE and MDA—critical biomarkers of oxidative stress that indirectly reflect the extent of lipid peroxidation—were significantly reduced following SeMC administration. Moreover, SeMC reduced the volume of cerebral infarction and attenuated BBB leakage in mice. In *in vitro* experiments, SeMC increased cell viability and maintained the barrier function of BMECs cells, whereas the protective effect of SeMC was accompanied by the inhibition of ferroptosis and the upregulation of TJ proteins. In conclusion, this study found that SeMC had a protective effect on IS by a mechanism attributed to the activation of the Akt/GSK3β pathway by SeMC and subsequent enhancement of downstream nuclear translocation of Nrf2 and β-catenin, which in turn increased the levels of GPX4 and TJ proteins, attenuated lipid peroxidation associated with ferroptosis, and maintained cellular viability of BMECs, thereby maintaining the integrity of the BBB. This suggests that SeMC shows promising potential in the treatment of stroke. The specific mechanisms by which existing research protocols can target and modulate ferroptosis in BMECs for the treatment of stroke are shown in (**Table 2**).

**Table 2:** Specific mechanisms for targeting and modulating ferroptosis in BMECs for the treatment of stroke.

Models	Animals/Cells	Target	Methods	Mechanisms	Ref.
MCAO	Male Wistar rats and BMVECs*	DFX	100 mg/kg, i.p.	AQP4↓,TNFα↓,ROS↓,ACSL4↓,LPO↓,HNE↓,GSH↑	(114)
MCAO	Female Wistar rats	DFX	100 mg/kg i.p.	AQP4↓,TNFα↓,NRF2↓,Cell survival↑	(115)
Acute hind limb I/R	Male C57BL/6 mice and HUVECs	FGF2	Overexpression	4-HNE↓,MDA↓,GSH↑,HO-1↑, 5-NQO1↑,SOD1↑,NFELE2↑	(125)
I/R and OGD/R	Male Wistar rats and bEnd.3 cells	p23	Overexpression	ROS↓,MDA↓,ACSL4↓,SLC7A11↓,GSH↑,GPX4↑,ZO−1↑	(136)
ICH and OGD/h	C57BL/6 mice and BMECs	METTL3	Silencing	Fe^2+^↓,ROS↓,LPO↓,MDA↓,TFR1↓,GPX4 m6A↓,GSH↑,GPX4 mRNA↑,SLC7A11↑	(142)
OGD/h	BMECs	LncRNA H19	knockdown	LPO↓,MDA↓,SLC7A11↓,TFR1↓,ACSL4 mRNA↓,GSH↑, GPX4↑,miR-106b-5p↑	(65)
dMCAO	C57BL/6 mice and TfR1^EC^ cKO mice	RosA-LIP	20 mg/kg/d, i.p.	DMT1(-ire)↓,DMT1(+IRE)↓,TFR1↓,ACSL4↓,ptgs2↓,LPCAT3↓,12-LOX↓,MDA↓,4-HNE↓,FTL↓,FTH↓,TJs↑,CD31↑,GSH↑,GPX4↑,SOD↑,CAT↑,NRF2↑,HO-1↑,FPN1↑	(170)
MCAO/R and OGD/R	C57BL/6J mice and bEnd.3	SeMC	2 mg/kg/d, i.p.	MDA↓,4-HNE↓,ACSL4↓,Claudin-5↑,GPX4↑,ZO-1↑,TEER↑	(179)

BMVECs*, BMVECs isolated from normal Wistar and type 2 diabetic Goto-Kakizaki rats; HUVECs, Human umbilical vein endothelial cells; OGD/h, oxygen and glucose deprivation hemin-treated; bEnd.3, Mouse-immortalized brain endothelial cells; i.p., intraperitoneal administration; i.g., intragastrical administration; ↓, Downregulated; ↑, Upregulated.

## 7 Discussion

In this review, we first comprehensively summarize recent research advances regarding the association between ferroptosis in BMECs and the pathological mechanisms of stroke. It has been established that ferroptosis in BMECs serves as a core pathological mechanism underlying stroke onset and progression, which mediates metabolic disorders of biochemical molecules through the regulation of multiple signaling pathways, thereby significantly exacerbating stroke progression. Consequently, targeted modulation of ferroptosis in BMECs may represent a crucial intervention strategy for neuroprotection following stroke. In contrast to the traditionally emphasized ferroptosis in neurons and glial cells, ferroptosis in BMECs holds unique pathological significance in BBB dysfunction following stroke. The disruption of microvascular endothelial integrity mediated by ferroptosis in BMECs leads to plasma protein extravasation and the formation of vasogenic brain edema. Additionally, the leakage of inflammatory mediators triggers neuroinflammatory cascades, while the trans-barrier transport of toxic substances induces subsequent brain injury. Furthermore, the imbalance in the coagulation-fibrinolysis system exacerbates the risk of hemorrhagic transformation. Consequently, this vascular unit-centered injury mechanism provides an innovative research perspective for elucidating the pathogenesis of post-stroke brain injury.

Secondly, this study further focused on the key molecular networks regulating ferroptosis in BMECs, focusing on resolving the following four signaling pathways: including Meg3/p53/GPX4 signaling pathway, TEAD1/MMP3 signaling pathway, SESN2/System Xc-/GPX4 signaling pathway and SP1/TNFSF9/SLC3A2 signaling pathway, these four signaling pathways are the regulatory hubs involved in ferroptosis in BMECs that have been clearly identified in current studies. Beyond that, Nrf2/HO-1 (180), HIF-1α/VEGF (181), PPARα-GOT1 (182), TLR2/4-MAPK (183), Serotonin/5-HT1A (184), AKT/mTOR (185), Ang-1/Tie-2 (186), ROBO4/VEGF (187), SIRT1/FOXO1 (188, 189), NOD2/MAPK/NF-κB (190), PPAR-γ/SIRT6/FoxO3 (191) and PI3K/Akt (192) signaling pathways are also involved in regulating BMECs, but whether these pathways can regulate ferroptosis in BMECs remains to be further validated.

After that, This review systematically summarizes therapeutic strategies targeting the ferroptosis in BMECs in stroke, untangling three major intervention dimensions: 1.Iron Homeostasis Regulation: The DFX suppresses post-stroke ferroptosis in BMECs by inhibiting the Fenton reaction and blocking lipid peroxidation cascades. 2.Genetic or Molecular Remodeling: Upregulation of FGF2 and p23 expression, METTL3 silencing, and LncRNA H19 knock-out effectively inhibit post-stroke ferroptosis in BMECs. 3.Novel Compound Intervention: RosA-LIP attenuate ferroptosis in BMECs by elevating GPX4 levels and reducing lipid peroxidation. Similarly, SeMC mitigates ferroptosis by enhancing GPX4 and tight junction protein expression, alleviating lipid peroxidation, and maintaining BMECs viability. These multimodal interventions significantly improve BBB integrity and reduce subsequent brain injury by targeting multiple pathways in post-stroke ferroptosis in BMECs. Notably, current research has shifted from single-pathway modulation to constructing multimodal regulatory systems. For instance, DFX combined with gene-editing technologies exhibits synergistic neuroprotective effects, while nanomaterial-based drug delivery systems (e.g., RosA-LIP) overcome the bioavailability limitations of conventional compounds. These breakthroughs not only provide innovative candidate therapies for stroke but also establish a theoretical foundation for neurovascular unit protection in the era of precision medicine. Future studies should prioritize spatiotemporal sequential interventions targeting multiple pathways, with dynamic efficacy evaluations using organoid models and *in vivo* imaging techniques.

Finally, some questions remain about the above studies: 1. Disease spectrum coverage: While this review primarily focuses on ferroptosis in BMECs in stroke, current investigations are predominantly limited to pathological models of ischemic and hemorrhagic stroke. Significant gaps remain in understanding the regulatory mechanisms and intervention strategies for ferroptosis in BMECs in specific stroke subtypes (e.g., subarachnoid hemorrhage). Establishing cross-species research platforms is warranted to systematically characterize molecular differences across stroke subtypes, elucidate the precise mechanistic roles of ferroptosis in BMECs in other stroke pathologies, and identify reliable treatment plans. 2. Translational medicine limitations: The existing evidence chain relies heavily on rodent experimental data, lacking validation through multicenter clinical cohorts or human organoid models. This substantially constrains the translational potential of basic research findings into clinical practice. 3. Incomplete clinical translation assessment framework: Pharmacokinetic profiles, Blood-Brain Barrier penetration efficiency, and long-term safety of ferroptosis-targeting agents (e.g., DFX and SeMC) developed for BMECs require further validation through clinical trials. 4. Insufficient mechanistic depth: The absence of specific molecular probes and conditional gene-editing models has delayed therapeutic target validation. Current research fails to establish direct causal relationships between ferroptosis in BMECs and clinical stroke outcomes.

## 8 Conclusion

In summary, ferroptosis in BMECs is a critical component in the pathological process of stroke. Investigating the relationship between ferroptosis and BMECs dysfunction may provide novel therapeutic targets for stroke. Based on this, this systematic review summarizes the biological functions of BMECs, the molecular mechanisms of ferroptosis, and their interactions in stroke, including relevant signaling pathways and potential treatment plans. These findings may offer a theoretical foundation for developing therapeutic strategies targeting ferroptosis in BMECs post-stroke. Furthermore, to address current challenges, future research should focus on constructing a multidimensional research framework, including: 1).Utilizing single-cell sequencing and spatial transcriptomics to map the ferroptosis-related molecular profiles of BMECs across different stroke subtypes; 2). Developing humanized BBB-on-a-chip and 3D organoid models to accelerate preclinical translational research; 3). Designing adaptive clinical trials to dynamically evaluate the efficacy-safety balance of ferroptosis-targeting drugs in stroke treatment; 4.Untangling the interaction network between ferroptosis in BMECs and other forms of programmed cell death through multi-omics integrative analysis. Only by achieving breakthroughs across the entire research continuum—from fundamental mechanistic exploration to clinical translation and precision medicine applications—can the clinical value of ferroptosis in BMECs as a novel therapeutic target for stroke be firmly established. This would enable effective modulation of ferroptosis in BMECs, alleviating stroke-induced brain damage and improving patient prognosis.
